# The global/local (limited to some regions) effect of cesarean delivery on the risk of pediatric allergic rhinitis: a systematic review and meta-analysis

**DOI:** 10.3389/fped.2023.1228737

**Published:** 2023-08-03

**Authors:** Xingyi He, Shipeng Zhang, Jiamin Wu, Qinwei Fu, Qinxiu Zhang, Wenyu Peng

**Affiliations:** ^1^ Hospital of Chengdu University of Traditional Chinese Medicine, Chengdu University of Traditional Chinese Medicine, Chengdu, China; ^2^ School of Clinical Medicine, Chengdu University of Traditional Chinese Medicine, Chengdu, China; ^3^ School of Medical and Life Sciences, Chengdu University of Traditional Chinese Medicine, Chengdu, China; ^4^ World Health Organization Collaborating Centre (WHOCC), Chengdu, China

**Keywords:** cesarean delivery, pediatric allergic rhinitis, risk factor, meta-analysis, children

## Abstract

**Background:**

Allergic rhinitis is a chronic and refractory disease that can be affected by a variety of factors. Studies have shown an association between cesarean section and the risk of pediatric allergic rhinitis.

**Methods:**

The PubMed, Springer, Embase, Cochrane Library, and Web of Science databases were searched to retrieve all studies published from January 2000 to November 2022, focusing on the relationship between cesarean section and the risk of pediatric allergic rhinitis. A meta-analysis was conducted to find a correlation between cesarean section and the risk of pediatric allergic rhinitis. A subgroup analysis was performed, considering the region and family history of allergy, after adjusting for confounding factors. Pooled odds ratios (ORs) were calculated, publication bias was assessed using a funnel plot, and heterogeneity between study-specific relative risks was taken into account.

**Results:**

The results showed that cesarean section was significantly associated with an increased risk of pediatric allergic rhinitis (OR: 1.27, 95% CI: 1.20–1.35). Subgroup analysis stratified by region indicated that cesarean section increased the risk of pediatric allergic rhinitis, with the highest increase in South America (OR: 1.67, 95% CI: 1.10–2.52) and the lowest in Europe (OR: 1.13, 95% CI: 1.02–1.25). The results of the subgroup analysis stratified by family history of allergy indicate that family history of allergy was not associated with the risk of pediatric allergic rhinitis.

**Conclusion:**

An association exists between cesarean section as the mode of delivery and the increased risk of pediatric allergic rhinitis, and cesarean section is a risk factor for allergic rhinitis.

## 1. Introduction

Allergic rhinitis is a chronic, non-infectious inflammatory response of the nasal mucosa caused by human exposure to allergens, manifested by symptoms such as itchy nose, sneezing, runny nose, and nasal congestion (1). The global average risk of allergic rhinitis has exceeded 20%, in which studies have reported that the proportion of children may be higher than that of adults (2), the majority of patients have symptoms before the age of 20 years, and nearly half of the patients develop symptoms at the age of 5–6 years in some countries (3, 4). Thus, the risk of pediatric allergic rhinitis is worthy of further attention. Medical therapy, specific immunotherapy, and surgery are the most commonly used methods for treating allergic rhinitis, while avoiding risk factors has always been the best option. In recent studies on risk factors for allergic rhinitis (3–5), cesarean section has been suggested as a risk factor for allergic rhinitis, and this method of delivery is significantly related to the risk of pediatric allergic rhinitis. Moreover, studies have shown that the rate of cesarean section is increasing (6–8), and the majority of countries remarkably exceed the reasonable rate of cesarean section presented by the World Health Organization (WHO) (10%–15%) (9). This finding is closely associated with the improvement of the socioeconomic level, the change in maternal health awareness, and the healthcare level (5, 10). The present systematic review and meta-analysis aimed to explore the relationship between cesarean section and the risk of allergic rhinitis in children, as the global demand for cesarean section has significantly increased. In addition, Cuppari et al. (11) found that cesarean delivery influences the risk of atopy but does not affect the prevalence of immune sensitization and allergic diseases.

Allergic rhinitis is a chronic and refractory disease that can be caused by a variety of factors. Studies have shown an association between C-sections and the risk of allergic rhinitis in children. In this study, systematic review and meta-analysis were conducted to investigate the relationship between cesarean section and the risk of allergic rhinitis in children. The results may guide pregnant women to choose their delivery methods, suggesting that choosing a natural delivery may reduce the risk of allergic rhinitis in children, especially in high-risk areas.

## 2. Methods

The protocol of this meta-analysis was prospectively registered at https://www.crd.york.ac.uk/prospero/#recordDetails(CRD42022378094) on 3 December 2022.

### 2.1. Search strategy of studies

The relationship between cesarean section and the risk of pediatric allergic rhinitis was explored. In this systematic review and meta-analysis, the articles published from January 2000 to November 2022 were searched using the PubMed, Springer, Embase, Cochrane Library, and Web of Science databases to assess the effects of cesarean section on the risk of pediatric allergic rhinitis. The use of keywords associated with medical subject terms (MeSH) cesarean section and allergic rhinitis was consistent with the preferred reporting items for systematic reviews and meta-analyses (Table 1). Specifically, three-word combined strategies were used for conducting the searches: Strategy 1: (cesarean OR caesarian OR cesarian OR C-section OR Caesarean delivery) AND (allergic rhinitis OR AR OR anaphylactic rhinitis); Strategy 2: (natural childbirth OR Spontaneous parturition OR spontaneous labor OR Natural Childbirth OR natural delivery) AND (allergic rhinitis OR AR OR anaphylactic rhinitis); and Strategy 3: Mode of delivery AND (allergic rhinitis OR AR OR anaphylactic rhinitis). Based on the title and abstract of the retrieved articles, their full text was searched if they analyzed the potential association between cesarean section and the risk of allergic rhinitis in children. Their reference lists were checked to search for similar studies before searching using the title and abstract. This process was repeated until no new references could be retrieved. The eligible studies should contain information about relative risk measures [e.g., unadjusted or adjusted odds ratio (aOR)], or information sufficient to calculate such measures (e.g., number of cases and controls based on the mode of delivery).

**Table 1:** The keywords associated with medical subject terms (MeSH) cesarean section and allergic rhinitis.

No.	Search terms
#1	Allergic rhinitis
#2	AR
#3	Anaphylactic rhinitis
#4	#1–#3/OR
#5	Cesarean
#6	Caesarian
#7	Cesarian
#8	C-section
#9	Caesarean delivery
#10	#–5–#9/OR
#11	Natural childbirth
#12	Spontaneous parturition
#13	Spontaneous labor
#14	Natural childbirth
#15	Natural delivery
#16	Eutocia
#17	#10–#15/OR
#18	Mode of delivery
#19	#4AND(#10OR#17OR#18)

The articles published from January 2000 to November 2022 were searched to assess the effects of cesarean section on the risk of pediatric allergic rhinitis using the PubMed, Springer, Embase, Cochrane Library, and Web of Science databases.

### 2.2. Study eligibility criteria

The inclusion criteria were as follows: (1) trials of allergic rhinitis in children and diagnosis of allergic rhinitis in accordance with international guidelines; (2) children aged 0–18 years and the availability of detailed statistics of types of childbirth and delivery methods; (3) cross-sectional, cohort, and case–control studies; (4) data that could be used to calculate 95% confidence intervals (CIs) for the effects of air pollutants on allergic rhinitis; and (5) trials published in the English language only, without the need of contacting the authors.

#### 2.2.1. Exclusion criteria

The exclusion criteria included incomplete data and data not accessible from the original author.

### 2.3. Data extraction

Data were initially extracted from the included studies. Then, two reviewers (XH and JW) independently extracted data from those studies, including the first author's name, year of publication, study duration, study type, sample size, diagnostic criteria, outcome evaluation criteria, and study results [data on the relative risk of allergy outcomes, including odds ratio (OR) and 95% CI, if available, and adjusted risk and risk of diagnosis/symptoms]. Discrepancies in the extracted data were resolved after a discussion with a third reviewer (SZ). Furthermore, the data were categorized by region (Asia, Europe, South America, and North America) and whether confounders could be adjusted for subsequent subgroup analysis.

### 2.4. Quality assessment

The quality scores used in the meta-analysis were found controversial. Thus, the quality of the included studies and the risk of bias were analyzed. Different assessment scales were used due to the different types of studies included. The included cohort and case–control studies were assessed using the Newcastle–Ottawa Scale (NOS), with a score of 14. Two reviewers (XH and JW) independently assessed the quality of the included cross-sectional studies using the Agency for Healthcare Research and Quality (AHRQ) scaling system (12), with scores of 0–3 indicating low quality, 4–7 moderate quality, and 8–11 high quality. Any disagreements were resolved through discussion with a third reviewer (SZ). Table 2 presents the results of the evaluation. Of the 21 articles included, one was excluded because of flaws in the study design, and another was excluded due to the large sample size and the serious effect on heterogeneity. Finally, the 19 remaining studies were analyzed, and the sensitivity analysis performed by SPSS software showed that after excluding most of the articles, the pooled results of the remaining studies were statistically significant (95% CI: 1), indicating that the results of the original meta-analysis were not easy to change significantly due to variations in the number of studies, and the results were therefore robust. Two subgroup analyses were also conducted.

**Table 2:** Information and quality of included literature.

Author	Years of publication	Country	Risk adjustment	Diagnostic methods of allergic rhinitis	The number of people included in the study	Allergic rhinitis OR (95% CI)	Quality score
Amélie Gorris MD	2020	Ecuador	Adjusted for sex, age, socioeconomic status, premature birth, exclusive breastfeeding, daycare attendance, father with allergy, born in Quito, and smoke exposure at home. Statistically significant values are presented in bold.	Standardized questionnaire from the International Study of Asthma and Allergies in Childhood project	400	0.99 (0.37–2.65)	5
Augusto Peñaranda	2011	Colombia	None	International Study of Asthma and Allergies in Childhood (ISAAC) questionnaires	3,256	1.40 (1.10–1.78)	5
Carlos Meza-López	2021	Mexico	None	International Study of Asthma and Allergies in Childhood (ISAAC) questionnaires	1,003	1.39 (0.78–2.48)	5
Doo Hee Han	2019	Korea	(1) plus duration of breastfeeding and number of siblings in the analysis using “mode of delivery;” (1) plus mode of delivery, and number of siblings in the analysis using “breastfeeding duration;” (1) plus mode of delivery, and duration of breastfeeding in the analysis using “number of siblings” (2)	All enrolled children received a physical examination and a skin prick test for a panel of 13 aeroallergens	1,374	1.25 (0.94–1.66)	8
Edyta Krzych-Falta	2018	Poland	None	ECRHS II and ISAAC questionnaires	18,617	1.20 (1.01–1.43)	6
Evelyn Xiu Ling Loo	2017	Singapore	Adjusted for maternal education level, paternal education level, family income, and maternal history of diabetes in pregnancy	Questionnaires were given at 3 weeks and 3, 6, 9, 12, 15, 18, 24, 36, 48, and 60 months	1,237	0.90 (0.60–1.35)	7
Heli Vieira Brandão	2016	Brazil	Adjusted for gestational age, weight at birth, breastfeeding up to the fourth month, mother's smoking during pregnancy, family income, mother's schooling level, parity, number of persons who sleep in the room with the child, attendance to the nursery to the age of 2 years, and pneumonia ever	International Study of Asthma and Allergies in Childhood (ISAAC) questionnaires	672	1.86 (1.18–2.93)	4
Jessica Gerlich	2016	Germany	None	Using questionnaires and clinical examinations	6,399	1.00 (0.40–2.50)	7
Ju-Hee Seo	2015	Korea	Adjusted for age, sex, parental income, parental history of allergic diseases, body mass index, exposure to tobacco smoke, and region	AR patients were defined as children who were diagnosed with AR by the physician using the questionnaire, and atopic AR patients as children who were diagnosed with AR by the physician using the questionnaire as well as by skin prick test results	1,828	1.07 (0.80–1.42)	7
McKeever TM	2002	UK	None	We identified each child (1) who was registered with a general practitioner within 3 months of birth and (2) whose medical history contained at least 1 consultation as an indicator that the child was using this general practitioner for his or her medical needs	24,690	1.01 (0.85–1.20)	6
Michael Pistiner	2008	USA	Adjusted for age and sex, in addition to all the variables listed in the column	A telephone questionnaire (modified from the American Thoracic Society–Division of Lung Disease Questionnaire) 19 was administered by trained research assistants to the child's primary caregiver until the age of 2 years	432	1.80 (1.00–3.24)	8
Montgomery SM	2000	UK	Adjusted for maternal age, prematurity (gestational age or birth weight), sibship (parity, birth order, or sibship size), parental allergy, and socioeconomic factor (parental education, level of income, or social class)	The data used here were collected at birth by midwives and by reference to medical records and, subsequently, at the ages of 5 and 10 years by interview (conducted by health visitors) and at the age of 26 years by a self-completed questionnaire	5,519	1.21 (0.84–1.74)	7
Nafstad P	2000	Norway	None	We conducted a follow-up study by using a self-administered questionnaire	2,531	1.20 (0.70–2.06)	7
Niki Mitselou	2019	Sweden	Adjusted for sex, maternal age at delivery, country of birth, parity, body mass index, early-pregnancy smoking, and maternal asthma/pulmonary disease	After being diagnosed by doctors, they were entered into the public health system	1,059,600	1.12 (1.08–1.16)	8
Peter Bager	2003	Denmark	Adjusted OR is adjusted for occupation and other variables in the table except ponderal index and SGA	Information on self-reported allergic rhinitis, asthma ever, current asthma, and occupation was obtained from 9,722 singleton women born in Denmark during the period of 1973–1977 who participated in a national cohort study during the period 1997–2001	9,722	1.16 (0.90–1.50)	6
Renz-Polster H	2005	USA	Adjusted for maternal age, prematurity (gestational age or birth weight), sibship (parity, birth order, or sibship size), smoking in pregnancy, and socioeconomic factor (parental education, level of income, or social class)	We searched the children's electronic medical records for diagnoses of asthma, allergic rhinoconjunctivitis (AR), atopic dermatitis (AD), and food allergies (FA) made at outpatient visits from 1996 to 2000	8,953	1.37 (1.14–1.65)	6
Salam MT	2006	USA	Adjusted for maternal age, prematurity (gestational age or birth weight), sibship (parity, birth order, or sibship size), parental allergy, smoking in pregnancy, and socioeconomic factor (parental education, level of income, or social class)	Information about sociodemographic factors, reported physician-diagnosed asthma, and other atopic diseases was obtained by using a self-administered structured questionnaire	3,464	1.57 (1.24–1.99)	7
Shuyuan Chu	2017	China	None	Parents were asked if the index child was ever diagnosed by a doctor as having asthma or allergic rhinitis	12,639	2.28 (2.00–2.59)	3
Xu B	2001	Finland	Adjusted for maternal age, prematurity (gestational age or birth weight), sibship (parity, birth order, or sibship size), parental allergy, smoking in pregnancy, and socioeconomic factor (parental education, level of income, or social class)	A clinical examination	12,058	1.28 (0.73–2.25)	6
Yeo Hoon Park	2010	Korea	Adjusted for gender, age, gestational age, birth weight, breastfeeding, and parental allergy	Data were extracted from medical records and a questionnaire filled out by parents	279	1.14 (0.61–2.10)	6
Youjin Li	2015	China	None	International Study of Asthma and Allergies in Childhood (ISAAC) questionnaires	20,803	1.36 (1.23–1.50)	4

### 2.5. Statistical analysis

The statistical analysis was performed using Review Manager (RevMan) 5.2 software (Cochrane Collaboration, London, UK). The OR value of 95% CI was calculated as the effect size. The Cochran *Q* test and Higgins *I*^2^ statistics were used to assess the level of heterogeneity between outcomes. The *Q* test and *I*^2^ statistics were utilized to examine heterogeneity, in which *I*^2^ > 50% indicated a significant heterogeneity. A fixed-effects or random-effects model indicated a significant difference in effect size (*P* < 0.05). The sensitivity analysis of the results and publication bias were evaluated using the leave-one-out and funnel plot techniques, respectively. A two-tailed *P *<* *0.05 was considered statistically significant.

## 3. Results

**Figure 1:**
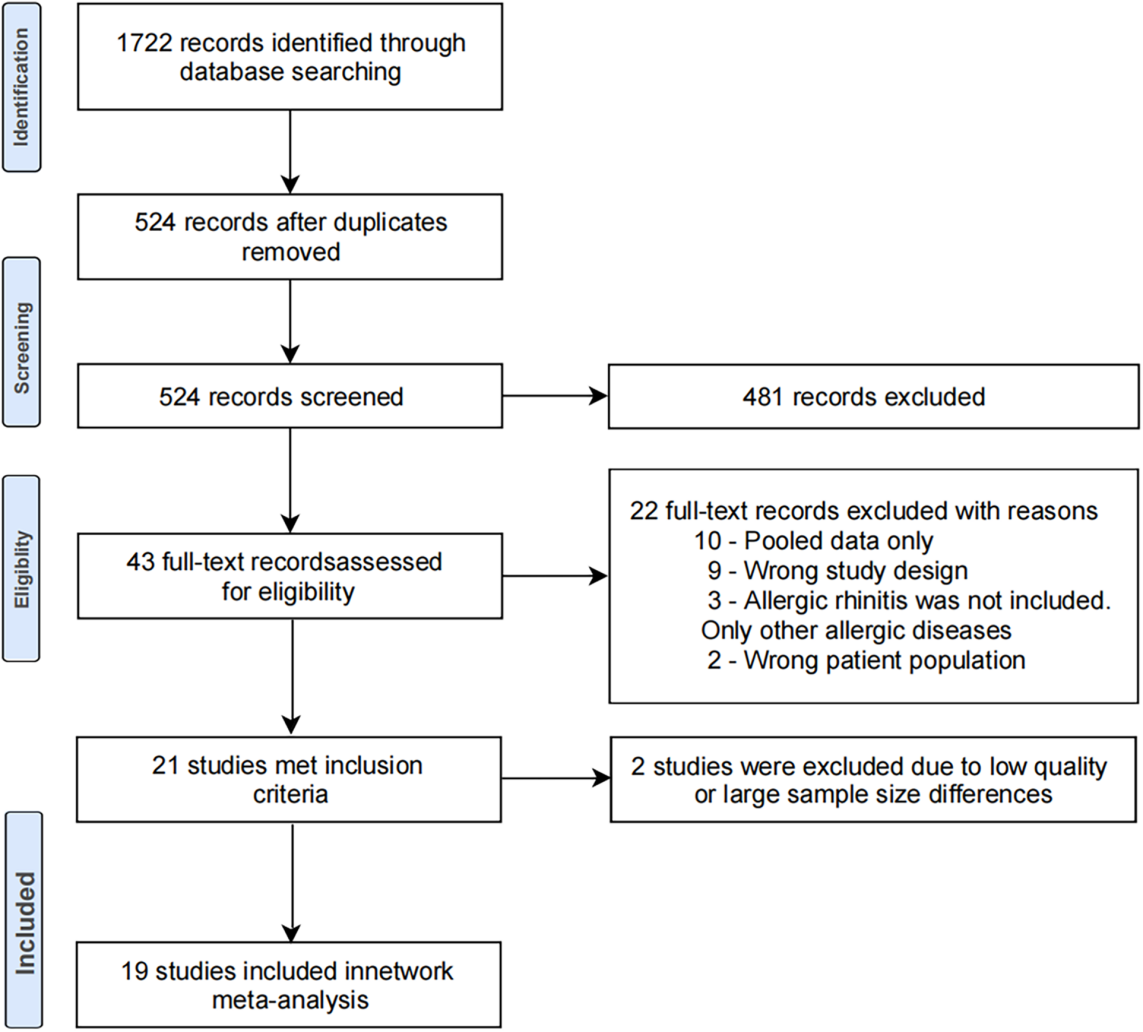
Preferred Reporting Items for Systematic Reviews and Meta-Analyses (PRISMA) flowchart.

Figure 1 shows the PRISMA flowchart for this meta-analysis. After searching through the proposed search strategy, a total of 1,722 articles were initially retrieved. After removing duplicate studies and reviews, 524 studies were obtained. Next, 481 articles were excluded due to irrelevance after evaluating their titles and abstracts, and the full texts of 43 studies were reviewed. Subsequently, 24 studies were excluded because they did not meet the inclusion criteria. As a result, 19 studies were included in the meta-analysis. These 19 studies were published in the English language, including 13 cohort studies and six cross-sectional studies. Among them, 10 studies directly examined the association between cesarean section or different modes of delivery and the risk of allergic rhinitis. Meanwhile, the other nine studies assessed the association between cesarean section or different modes of delivery and allergic disease or asthma, including allergic rhinitis.

### 3.1. Meta-analysis

**Figure 2:**
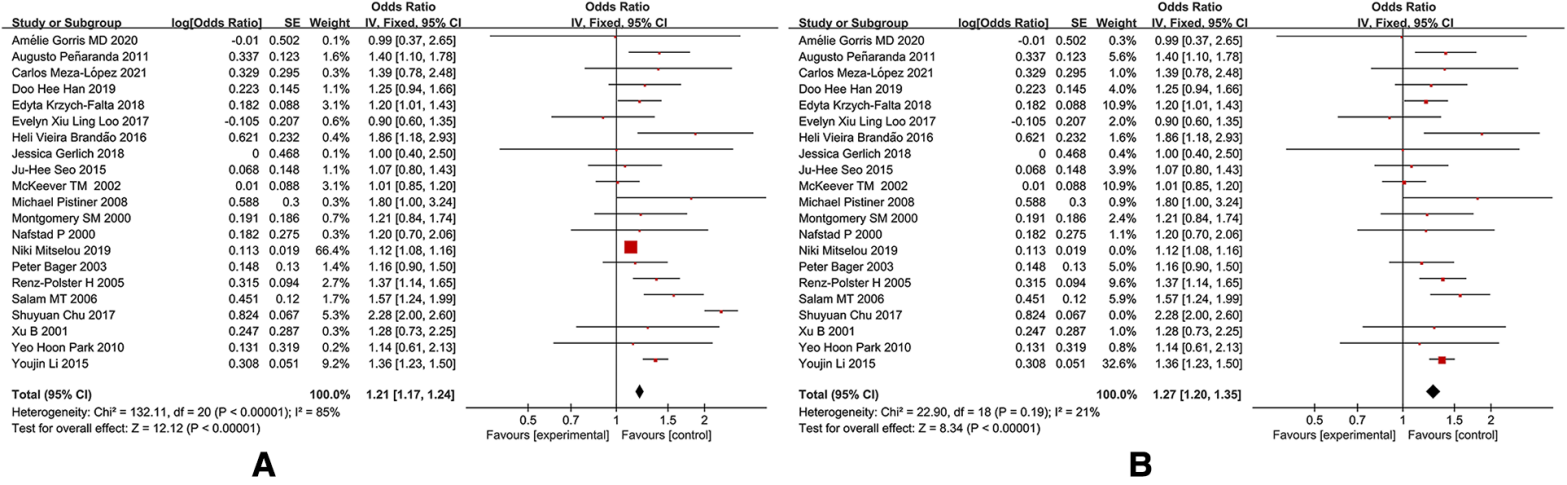
A forest plot of cesarean section and AR prevalence in children. (A) Cesarean section and allergic rhinitis in children after 21 articles were included. (B) Cesarean section and allergic rhinitis in children after 2 articles were excluded.

Figure 2 shows the relationship between cesarean section and the risk of pediatric allergic rhinitis in the 19 included studies. Three non-compliant studies were excluded after conducting a quality assessment and sensitivity analysis. The final results showed that cesarean section was significantly associated with the risk of allergic rhinitis in children, and cesarean section was associated with an increased risk of allergic rhinitis in children (OR: 1.27, 95% CI: 1.20–1.35).

#### 3.1.1. Subgroup analysis by region

**Figure 3:**
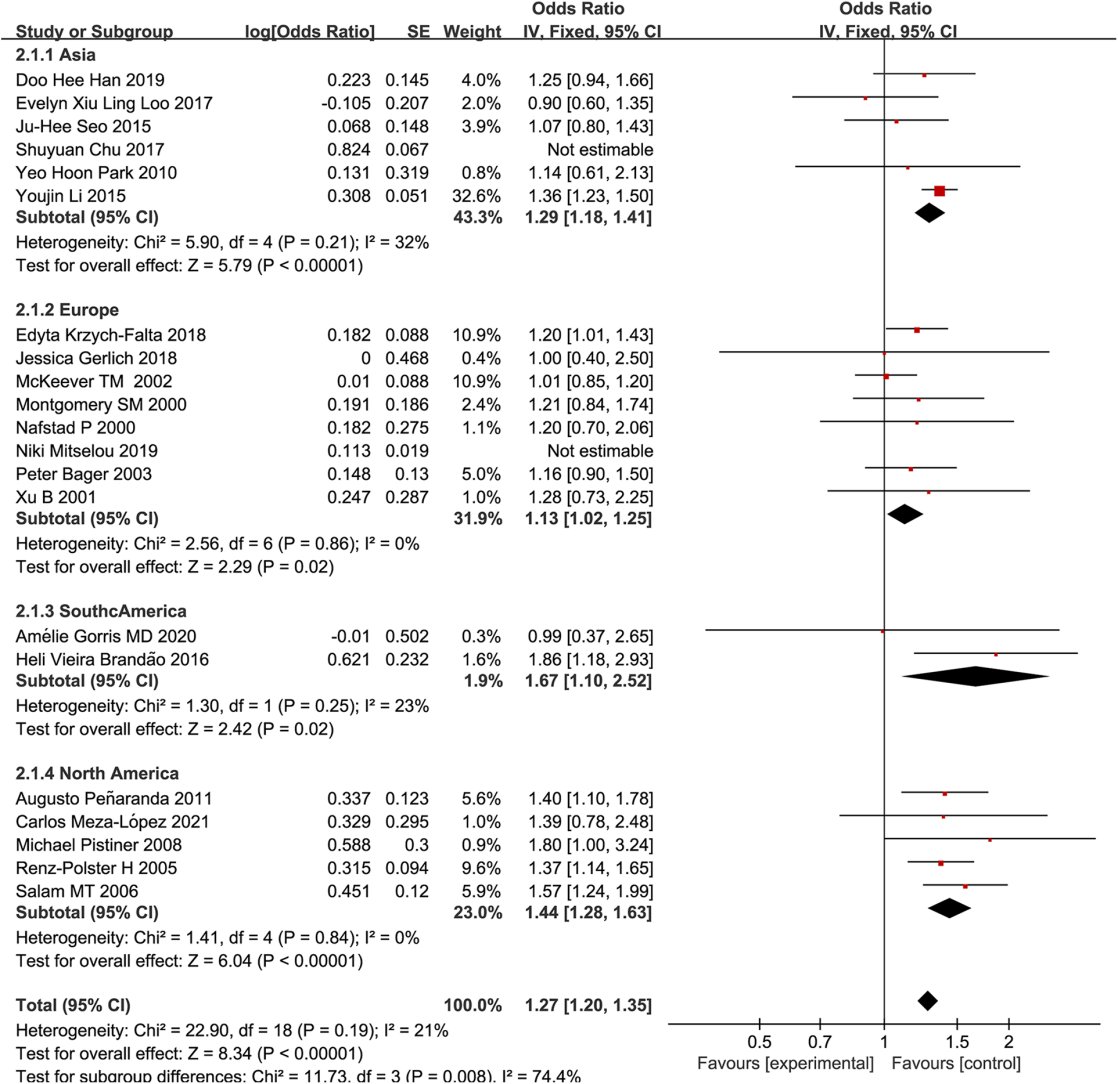
A forest plot of cesarean section and child AR prevalence in different regions.

The results of subgroup analysis by region (Asia, Europe, South America, and North America) showed that the risk of allergic rhinitis in children delivered by cesarean section was most increased in South America (13, 14) (OR: 1.67, 95% CI: 1.10–2.52), followed by North America (15–19) (OR: 1.44, 95% CI: 1.28–1.63), Asia (5, 20–23) (OR: 1.29, 95% CI: 1.18–1.41), and Europe (10, 24–29) (OR: 1.13, 95% CI: 1.02–1.25), as shown in Figure 3.

#### 3.1.2. Subgroup analysis by family history of allergy

**Figure 4:**
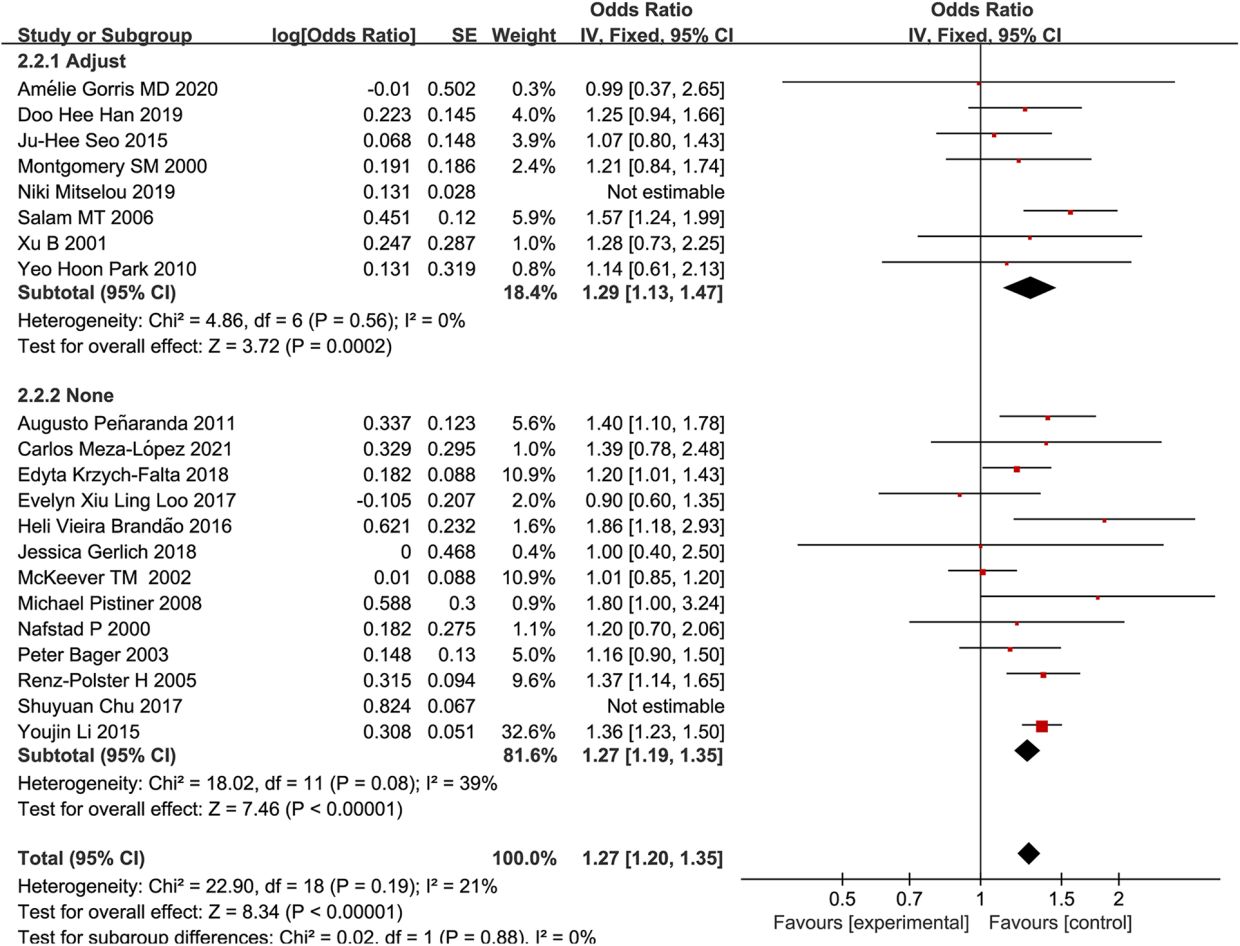
A forest plot adjusting for family allergy history confounding factors after cesarean section and the prevalence of AR in children.

A subgroup analysis based on whether the confounders were adjusted was performed. It was revealed that the overall OR of studies that did not adjust for confounding factors for family history of allergy (OR: 1.27, 95% CI: 1.19–1.35) did not significantly differ from the overall OR of studies that adjusted for confounding factors for family history of allergy (OR: 1.29, 95% CI: 1.13–1.47), as shown in Figure 4, suggesting that there was no significant relationship between family history of allergy and the risk of pediatric allergic rhinitis. This finding contradicts the conclusion in some studies that family history of allergy increased the risk of pediatric allergic rhinitis.

### 3.2. Publication bias

**Figure 5:**
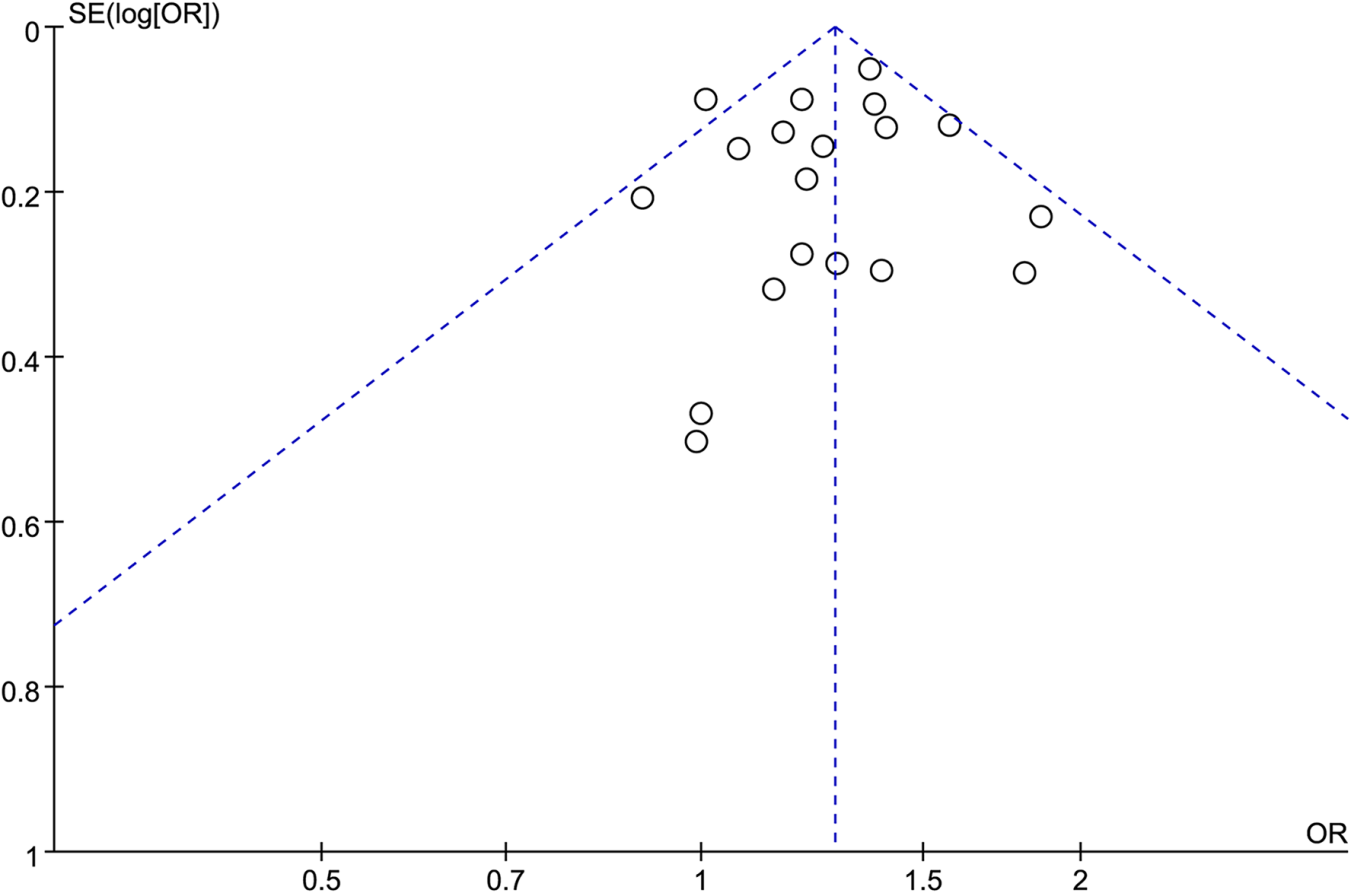
Funnel plots (publication bias of included literature).

We analyzed the publication bias of 19 studies on the relationship between cesarean section and AR risk in children using a funnel plot (Figure 5). The results show that the points are basically symmetric and distributed at the top of the funnel, but one report by McKeever et al. (10) was distributed outside the funnel plot, which may be due to the absence of confounding adjustment or late follow-up.

## 4. Discussion

Allergic rhinitis is a respiratory disease that can be affected by several factors. This meta-analysis examined the relationship between cesarean section and the risk of pediatric allergic rhinitis. As a result, the disorder of physiological bacterial colonization in children, especially in the gastrointestinal tract, affects the immune system (30), hinders the transformation of neonatal Th2 to Th1-biased T cell memory (31), and thus causes an increased risk of allergic diseases. Several studies on cesarean section have shown that delivery via cesarean section might increase the risk of allergic diseases, such as allergic rhinitis, asthma, eczema, and food allergies, similar to the results of the present study. However, this was diametrically opposed by the findings of Gorris et al. (13, 21, 32, 33), who demonstrated that no significant relationship exists between delivery via cesarean section and the risk of pediatric allergic rhinitis. The effect of delivery via cesarean section on allergic rhinitis has remained controversial in recent years, and the differences might be attributed to insufficient sample sizes, dietary differences, weather differences, and exposure levels, indicating the necessity for further research.

The present study analyzed the association between cesarean section delivery patterns and the risk of pediatric allergic rhinitis after adjusting for confounding factors, such as age, gender, birth weight, passive smoking, feeding patterns in the first 6 months, maternal educational level, household income, and antibiotic exposure. However, due to lacking raw data, adjusting for confounding factors for maternal diseases during pregnancy and family allergy was infeasible. Mitselou et al. (34) categorized the causes of cesarean section into elective labor and emergency delivery and showed that emergency cesarean section increased the risk of allergic rhinitis more than optional cesarean section after adjusting for confounding factors. Emergency cesarean section was mainly associated with maternal morbidity during pregnancy, and some studies have found that pregnancy-associated complications increased the risk of allergic diseases in children and the risk of intrauterine infection (35). Pistiner et al. (17) analyzed study subjects with and without family history of allergy, and the results showed that in children with family history of allergy, cesarean section increased the incidence of allergic rhinitis. In the present study, the included studies were divided into two groups based on whether they adjusted for factors for family history of allergy or not, and a subgroup analysis was performed. It was found that the risk of pediatric allergic rhinitis in studies that adjusted for factors for family history of allergy was OR: 1.29, 95% CI: 1.13–1.47, which was not significantly different from those studies that did not adjust for factors for family history of allergy (OR: 1.27, 95% CI: 1.19–1.35). This result, contrary to recent studies that demonstrated that family history of allergy had an influence on the risk of pediatric allergic rhinitis (17, 25, 36), is worthy of further assessment using more research to verify whether allergic genetic predisposition can interact with the effects of cesarean section, making allergic rhinitis more likely to occur in children.

In the present study, subgroup analysis was performed on data stratified by region. For this purpose, four subgroups of Asia, Europe, South America, and North America were considered. The results of subgroup analysis revealed that in Asia (OR: 1.29, 95% CI: 1.18–1.41), Europe (OR: 1.13, 95% CI: 1.02–1.25), South America (OR: 1.67, 95% CI: 1.10–2.52), and North America (OR: 1.44, 95% CI: 1.28–1.63), both cesarean delivery methods increased the risk of pediatric allergic rhinitis, while OR indicated a different level of risk. The results showed that the risk of pediatric allergic rhinitis was the highest in South America and the lowest in Europe. ECRHS reports have also shown that the risk of nasal allergies varies by geographical region (37), which may be related to regional climate and topographic differences, dietary structure, degree of industrialization, etc. (35, 38, 39).

Europe is located in the northeastern hemisphere and has rich fishery resources, and fish has become a very important part of the daily diet. South America is located in the western and southern hemispheres, and due to the climatic and environmental conditions, its animal husbandry is more developed, and the local people mainly consume beef products. Fish has a richer vitamin D content than beef. Previous studies have shown that allergic rhinitis is associated with a reduced vitamin D level (38, 40–42), which can speculate that the European’s preference for eating fish has significantly reduced the risk of allergic pediatric rhinitis. Testa et al. (35) have studied a large number of studies and found that these studies had taken numerous factors into account in a large and representative population, provided that a relationship between dietary nutrition and allergic rhinitis, and improved pediatric eating habits may reduce the risk of allergy symptoms. This study revealed that due to the different dietary patterns in different regions of the world, pediatric intake of nutrients differs, which may result in different incidence rates of allergic rhinitis.

South America is located almost between the equator and the Tropic of Capricorn, and its climate is mainly tropical rainforest. Compared with the climate of Europe, the climate of South America is characterized by high temperature, high humidity, and heavy rainfall. Such climatic characteristics have led to differences in mite species and concentrations in different regions, especially in the Andes of Colombia, Peru, and Venezuela, where the prevalence of mites is high (43, 44), and mites are a very important risk factor for allergic rhinitis. Cingi et al. (38) demonstrated that although the overall level of the atopic epidemic did not increase due to climatic factors, climatic changes were related to the variations in the geographical patterns of the atopic epidemic. They also found that the differences in climate between South America and Europe can lead to different effects of cesarean section on the risk of pediatric allergic rhinitis.

The risk of pediatric allergic rhinitis from cesarean section is also associated with the degree of industrialization and development of the region. Chou et al. (12) assessed the risk of cesarean section and allergy in children and found a significantly higher risk than that reported in highly industrialized countries. This finding may be related to specific conditions, and the degree of industrialization of a region mainly affects a country's degree of development, economic level, healthcare level, air pollution (45, 46), etc. These factors may influence the maturity of cesarean section, the level of healthcare during production, etc., in the region, resulting in different risk levels in different regions of the world.

The high level of industry in Europe, combined with robust healthcare, a great climate, and a healthy diet containing vitamin D, may explain why cesarean section in Europe is associated with a lower risk of pediatric allergic rhinitis compared with that in other regions.

## 5. Strengths and limitations

To our knowledge, this study is one of the few recent meta-analyses that have studied the relationship between cesarean section and the risk of pediatric allergic rhinitis, and the results were meaningful for both the prevention and selection of delivery methods for pediatric allergic rhinitis. However, the results indicate the necessity of conducting further relevant research. It is noteworthy that the number of relevant studies was limited; thus, additional studies should be performed to clarify the differences in the risk of pediatric allergic rhinitis stratified by region. Moreover, the number of research subjects in the included studies remarkably varied. In the present meta-analysis, the majority of the included studies were cross-sectional studies, and patients had recall biases in their memories, demonstrating the necessity of more cohort studies to supplement the findings. At the same time, it is well known that prophylactic antibiotics are usually used to avoid infection after cesarean section, and antibiotic use also exists after some vaginal births. We found that this detail was not documented in the present study, so this factor may influence our analysis results. We need to conduct a long-term cohort study in the future to observe whether using antibiotics after delivery has a certain influence on the early development and health of children.

## 6. Conclusions

In summary, the results of the present meta-analysis suggested that the mode of delivery via cesarean section increased the risk of pediatric allergic rhinitis after adjusting for confounding factors. The results of the subgroup analysis showed that children in high-risk areas were more affected by cesarean section. In contrast, our experimental results did not support the assumption that family history of allergy increased the risk of pediatric allergic rhinitis. The results of this study provide some reference value for pregnant women in choosing their delivery method, especially in high-risk areas. However, the final choice of pregnant women is influenced by many factors, and the final mode of delivery should be combined with individual specific circumstances. Further evidence is required to clarify the relationship between family history of allergy and the risk of pediatric allergic rhinitis. However, the sample size of the present study was not large enough, it was a retrospective study, and the adjustment of confounding factors was insufficient. Thus, additional studies are required to verify the conclusions.
